# Deficiency of mast cells in coronary artery endarterectomy of male patients with type 2 diabetes

**DOI:** 10.1186/1475-2840-10-40

**Published:** 2011-05-14

**Authors:** Aleš Pleskovič, Olga Vraspir-Porenta, Ruda Zorc-Pleskovič, Danijel Petrovič, Metka Zorc, Aleksandra Milutinović

**Affiliations:** 1 University Medical Centre of Ljubljana, Department of Internal Medicine, Zaloška 2, 1000 Ljubljana, Slovenia; 2 Medical Faculty, University of Ljubljana, Institute of Histology and Embryology, Korytkova 2, 1000 Ljubljana, Slovenia

## Abstract

**Background:**

Type 2 diabetes is an important risk factor for the development of coronary artery disease (CAD). Focal or diffuse inflammation is often present in the vessels of patients with CAD. Mast cells are frequently present in the plaques as well as in the inflammatory infiltrates in the atherosclerotic vessel wall. In the study we wanted to examine whether there are differences in the morphology, number and distribution of mast cells and in their ability to modify the atherosclerotic process in coronary arteries (CA) in the diabetic *vs*. the hypertensive population of patients with CAD.

**Methods:**

Coronary artery endarterectomy specimens were obtained from patients with diabetes or hypertension as the only risk factor for CAD. The specimens were stained with haematoxylin-eosin and Sulphated Alcian Blue for mast cells and with immunofluorescent methods for fibrinogen-fibrin and IgG deposits in the vessel wall. Both morphological and stereological assessments were conducted for mast cells and mononuclear cell infiltrates.

**Results:**

The histological analysis of the vessel wall of diabetic patients in comparison with hypertensive patients showed a damaged endothelial cells layer and deposits of fibrin-fibrinogen and IgG in the tunica intima and media. The stereological count revealed a diminished numerical density of mast cells and a significantly higher volume density of the mononuclear cells. Mast cells displayed cytoplasmic vacuolization, extracellular extrusion of granule and pyknotic nuclei.

**Conclusion:**

This preliminary study suggests that the impaired mast cells might be the reason for more extensive inflammatory and immunologic atherosclerotic changes in the CA vessel wall of CAD patients with type 2 diabetes.

**Trial registration:**

134/88;C3-0564-381-92

## Background

Coronary artery disease (CAD) can be macroscopically visible as one or more localized atherosclerotic plaques, or as a diffuse, long, concentric thickness of the vessel wall, which protrudes and obstructs the vessel lumen. These changes alter the structure of the vessel wall, ultimately disrupting normal cardiac function. CAD is defined as a chronic inflammatory disease, and several cytokines and growth factors are involved in the pathogenesis of the disease [1-6]. Surgical procedures on coronary arteries (endarterectomy) enable the performance of morphologic and morphometric analyses of atherosclerotic changes of the coronary arteries. Important risk factors for the development of CAD are type 2 diabetes and arterial hypertension. Atherosclerosis is now generally accepted as a chronic inflammatory condition and there is also growing evidence of an important role of chronic inflammation in type 2 diabetes [7].

The differences in the content and recruitment [8] of various inflammatory cells [9] and their autocrine and paracrine secretory activities have been reported to influence the fate of the atherosclerotic plaque. Among the inflammatory cells, mast cells are obligatory accompanying elements of the localized and diffuse inflammatory changes in the atherosclerotic vessel wall. In the last few years, several investigations have shown that the largest density of mast cells is located in the atherosclerotic plaque of the vessel wall, both in early and advanced CAD, especially in the shoulder region of unstable plaques [10-12]. Mast cells play a crucial role in the inflammatory process [13,14]*via *many secretory mediators [15], in the activation of other inflammatory cells (e.g. lymphocytes, T macrophages and foam cells) and in influencing the metabolism and the circulation of HDL and LDL lipoproteins. Proteoglycans and proteases derived from activated mast cells play an important role in the regulation of coagulation and fibrinolysis, processes that are closely connected with the development and complications of the atherosclerotic process. The release of heparin as an anticoagulant substance from the mast cells, which leads to higher endogenous heparin levels and higher levels of IgE, may have the primary role in the protective function of vessel wall endothelial cells and act to reduce the complications of CAD [16]. It was reported that [17] mast cells may influence the course of the atherosclerotic process by releasing cytokines from their secretory granules and by coordinating the transportation of inflammatory cells in the vessel wall.

We hypothesized that the morphology, number, distribution and probably the function of mast cells may differ in CAD patients with type 2 diabetes or arterial hypertension, as the only risk factor. Thus, we examined endarterectomy specimens of CA from CAD patients with diabetes or with arterial hypertension using histological and histomorphometrical analyses (a stereological count with the volume density of the mononuclear infiltrates, and the numeric density of mast cells).

## Methods

Coronary endarectomy sequesters were obtained during Coronary Artery Bypass Graft Surgery (CABG) at the University Clinic of Cardiovascular Surgery, Novi Sad, Serbia, from 20 patients with CAD. The study was approved by the National Medical Ethics Committee. In 20 patients with CAD CABG surgery was performed due to angina pectoris, whereas endarectomy was performed in those coronary arteries with diffuse stenotic lesions. Twenty consecutive patients with diffuse CAD were enrolled in the study with either type 2 diabetes or arterial hypertension. The exclusion criteria were type 1 diabetes and the presence of both disorders (arterial hypertension and type 2 diabetes). Patients were divided in two groups the group of patients with type 2 diabetes only (N = 10) and the group of patients with arterial hypertension only (N = 10) (Table 1). The diabetic patients had been treated for 9.5 ± 1.716 years, the hypertensive patients for 7.5 ± 1.080 years. After informed consent was obtained from the patients, a detailed interview was made. Patients were classified as having type 2 diabetes according to the current American Diabetes Association criteria for the diagnosis and classification of diabetes [18]. The body mass index (BMI) was calculated as weight in kilograms divided by height in square meters. Total cholesterol, low density lipoproteins (LDL), high density lipoproteins (HDL) and triglycerides were determined by standard biochemical methods. Cigarette smoking was defined as a binary variable.

**Table 1:** Clinical data about diabetic and hypertensive groups of patients

Characteristics	Diabetic group	Hypertensive group	P value
Number	10	10	
Age (years)	56.6 ± 7.58	60 ± 6.16	0.274
BMI (kg/m^2^)	28.08 ± 2.44	27.6 ± 3.37	0.72
Systolic pressure (mmHg)	126.5 ± 6.26	174.5 ± 17.07	< 0.001
Diastolic pressure (mmHg)	79.5 ± 5.50	113.4 ± 10.80	< 0.001
Glucose level (mmol/l)	7.54 ± 1.61	4.64 ± 0.82	< 0.001
Total cholesterol (mmol/l)	6.52 ± 1.57	5.82 ± 1.56	0.331
HDL cholesterol (mmol/l)	1.04 ± 0.04	1.25 ± 0.45	0.037
LDL cholesterol (mmol/l)	3.26 ± 1.43	3.85 ± 1.68	0.41
Triglycerides (mmol/l)	2.35 ± 0.76	1.78 ± 0.6	0.856
Cigarette smoking	2 (20%)	3 (30%)	0.65

**Figure 1:**
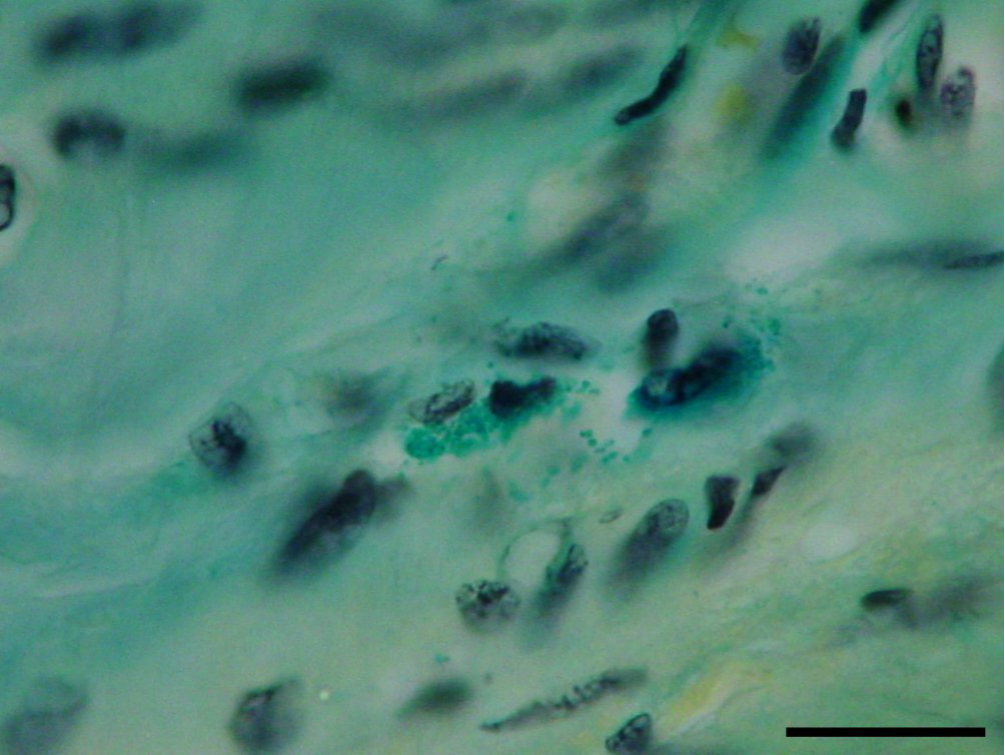
**Degranulated mast cells with picnotic nucleus (bar = 20 μm)**.

Endarterectomy specimens were surgically taken from the left anterior descendent coronary artery, the left circumflex coronary artery, or the right coronary artery. The tissue samples were formaldehyde fixed, embedded in paraffin and cut into 5 μm thick serial sections. The standard haematoxylin-eosin staining method for the vessel wall tissue and the Sulphated Alcian Blue method for the demonstration of intracellular or extracellular localization of mast cell granules were used. Immunofluorescent methods were performed to show fibrinogen-fibrin and IgG deposits in the endarterectomy specimens as described previously [19]. The number of mast cells and mononuclear cells were counted in 20 random high power fields (× 400). The numerical density of the mast cells and the volume density of the mononuclear cells infiltrates were calculated using previously described stereologic methods [20,21]. Histological, immunofluorescent and stereological analyses were performed in step serial sections. Degranulated mast cells were defined as cells with > 50% of cytoplasmic granules extruded extracellular in the vicinity of the cell body (Figure 1). Their presence was noted in 20 random high power fields (× 1000 magnification) [22]. Numerical data are reported as a mean value ± SD. The stereological results for mast cells and mononuclear cells infiltrates for diabetic and non diabetic patients were analyzed with the Student t-test. The *P *value < 0.05 was considered as statistically significant. A statistical analysis was performed using the Excel program, Microsoft Office 2003.

## Results

The clinical data of patients with type 2 diabetes and arterial hypertension are shown in Table 1. Patients had no statistically significant difference in BMI, total cholesterol, LDL cholesterol and triglycerides, and in the incidence of cigarette smoking, whereas the difference in the HDL cholesterol level was of borderline significance. Due to different inclusion criteria, they differ in blood pressure and glucose level (Table 1).

Moreover, they did not differ in the frequency of lipid-lowering drugs, beta blockers, ACE inhibitors and antiplatelet drugs.

In diabetic patients, a histological analysis revealed a focal or diffuse mononuclear cells infiltration, the presence of fibrin-fibrinogen and IgG deposits in the tunica intima and tunica media of the endarterectomized coronary vessels. In hypertensive patients, fibrin-fibrinogen as well as IgG deposits were absent, and mononuclear infiltrates were rarely observed. In CAD patients with type 2 diabetes, damaged endothelial cells and large areas of luminal boundaries without endothelium with denuded intimal layers were found. In CAD patients with arterial hypertension only, continuous endothelial cell layers were demonstrated.

**Figure 2:**
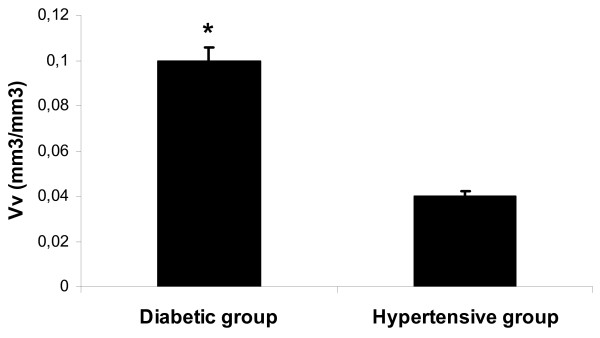
**Volume density (Vv) of mononuclear cells infiltrates in diabetics in comparison with hypertensive patients**. *P < 0.05.

**Figure 3:**
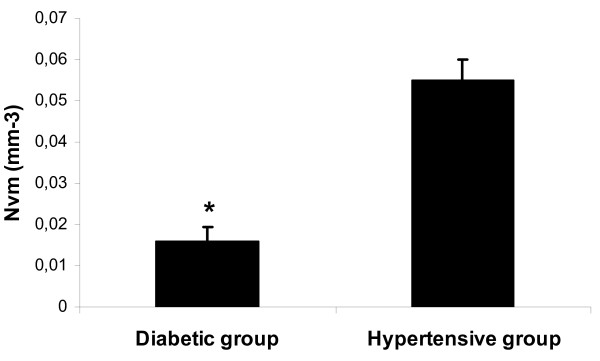
**Numerical density of mast cells (Nvm) in diabetic group in comparison with hypertensive group of patients**. * *P *< 0.05.

The morphological analysis of mast cells showed that the nuclear pyknosis, vacuolization and degranulation of mast cells were more intense in diabetic patients in comparison with hypertensive patients. Morphometric analysis demonstrated a significantly higher volume density (Vv) of mononuclear cells infiltrates in diabetic patients in comparison with hypertensive patients (Figure 2). Moreover, a lower numerical density of mast cells (Nvm) was demonstrated in diabetic patients in comparison with non-diabetic patients with arterial hypertension (Figure 3).

## Discussion

In this preliminary study we demonstrated a clear difference in atherosclerotic lesions in CAD patients with type 2 diabetes in comparison with the patients from the hypertensive group. The samples from diabetic patients showed a greater degree of inflammation compared to the samples of hypertensive patients. The fibrin-fibrinogen and IgG deposits, accompanying abundant mononuclear cells infiltrates, and damaged endothelial cells of coronary arteries may indicate that atherosclerosis in diabetic patients constitutes humoral [23] and cellular immune responses [24,25]. We postulate that normal mast cells play an important role in the protection of the integrity of endothelial cell layers and the vessel wall, probably *via *their paracrine activity, especially through the release of heparin. It is known that in hyperglycemic conditions heparin may protect cells from injury [26]. In diabetic patients, the nuclear pyknosis, degranulation and vacuolization of the mast cells in the CA indicate that they are damaged. Therefore, their protective role of the vessel wall may be diminished. Their impairment, lower numeric density, nuclear pyknosis, degranulation and vacuolization of the mast cells in diabetic patients might be the reasons for more extensive inflammatory and immunologic atherosclerotic changes observed in diabetic CAD patients in comparison with hypertensive CAD patients. The lower number of impaired mast cells cannot coordinate the normal transportation of mononuclear cells through the vessel wall. Inflammatory cells, fibrin-fibrinogen and IgG deposits accumulate in the vessel wall as atherosclerotic plaques that protrude and occlude vessel lumen. In contrast, in hypertensive CAD patients, the morphology of mast cells was normal and the inflammatory changes were less intense. Fibrin-fibrinogen and IgG deposits accumulations were absent. A diminished numerical density of impaired mast cells and a significantly higher number of mononuclear cells infiltrates in the atherosclerotic plaques of CA vessel wall indicate differential inflammatory processes within the CA vessel wall in diabetic patients *vs*. hypertensive patients.

A major limitation of this preliminary study is the relatively low number of CAD cases with either type 2 diabetes or arterial hypertension. We are planning to enroll new CAD cases with either type 2 diabetes or arterial hypertension to increase the reliability of the study. However, there are only some patients with CAD suitable for the endarterectomy procedure. Further studies enrolling larger numbers of CAD are needed to confirm our findings.

## Conclusion

This preliminary study suggests that the impaired mast cells might be the reason for more extensive inflammatory and immunologic atherosclerotic changes in the CA vessel wall of CAD patients with type 2 diabetes. Moreover, we speculate that mast cells are differently involved in atherosclerotic lesions in CAD patients with type 2 diabetes in comparison with hypertensive patients.

## Abbreviations

CAD: coronary artery disease; CA: coronary arteries; BMI: Body mass index; HDL: high density lipoprotein; LDL: low density lipoprotein; IgG: immunoglobulin G; Vv: volume density; Nvm: numerical density of mast cells; SD: standard deviation;

## Competing interests

The authors declare that they have no competing interests.

## Authors' contributions

AM designed, coordinated and wrote the manuscript. AP and MZ provided the human coronary arteries and wrote the manuscript. DP led the treatment of patients and wrote the manuscript. AP, RZP and OVP made the histological examination, stereological and statistic analyses and wrote the manuscript. All authors have read and approved the final manuscript.
